# A197 A ROLE FOR NOD2 IN INTESTINAL T CELL MEMORY DEVELOPMENT AND FUNCTION

**DOI:** 10.1093/jcag/gwad061.197

**Published:** 2024-02-14

**Authors:** B K Tsankov, A Luchak, N Nathan, D Philpott

**Affiliations:** Immunology, University of Toronto, Toronto, ON, Canada; Immunology, University of Toronto, Toronto, ON, Canada; Immunology, University of Toronto, Toronto, ON, Canada; Immunology, University of Toronto, Toronto, ON, Canada

## Abstract

**Background:**

Aberrant resident memory T cell (TRM) responses have been associated with increased intestinal inflammation and Crohn’s disease (CD) pathology in humans. Intestinal TRM cells are not only important for maintaining the integrity of the intestinal epithelial barrier, but also for the rapid clearance of pathogens in the intestine during infection. Understanding the signals received by the intestinal immune system to generate TRM responses is paramount to elucidating treatments for CD. Genetic mutations in NOD2 are associated with the highest risk of CD development. As a host intracellular sensor of bacterial peptidoglycan, NOD2 is critical for initiating both innate and adaptive immune responses. Furthermore, work from our lab as well as those of our collaborators suggest that NOD2 deficiency reduces systemic memory B and T cell responses. However, the role of NOD2 in establishing memory T cell responses in the intestine remains unclear. This work will therefore establish the role of NOD2 signaling in initiating and maintaining optimal TRM responses to achieve intestinal homeostasis and resilience to intestinal inflammation.

**Aims:**

It is the main objective of this project to determine whether NOD2-mediated signalling affects:

1. Antigen-specific intestinal T cell priming in vivo

2. Bona fide intestinal TRM generation

3. Bona fide intestinal TRM function

**Methods:**

Intraperitoneal LCMV-Armstrong infection results in long-lived CD4^+^ and CD8^+^ resident-memory T cell generation in the intestinal lamina propria of mice. Wildtype and NOD2 deficient littermate mice were infected with LCMV-Armstrong, and the effects on intestinal T cell priming, effector function, and memory T cell function were examined by flow-cytometry, *ex vivo* and *in vivo* antigen-recall experiments, qPCR, and viral plaque assays.

**Results:**

Augmented NOD2 signalling during priming increased LCMV-specific CD4^+^ T cell numbers in the mesenteric lymph nodes - but not in the spleen, independent of proliferation or cell death. Furthermore, NOD2-deficiency resulted in decreased effector and memory T cell numbers in the small intestinal lamina propria. This effect was associated with decresead memory CD4^+^ and CD8^+^ T cell functions in NOD2-deficient mice during *ex vivo* and *in vivo* peptide recall experiments.

**Conclusions:**

NOD2 affects T cell priming in the mesenteric lymph nodes, which later affects effector and memory T cell functions in the intestinal lamina propria.

**Funding Agencies:**

CAG, CIHREmerging and Pandemic Infections Consortium

